# Crystal structure of the catalytic core of Rad2: insights into the mechanism of substrate binding

**DOI:** 10.1093/nar/gku729

**Published:** 2014-08-12

**Authors:** Michał Miętus, Elżbieta Nowak, Marcin Jaciuk, Paweł Kustosz, Justyna Studnicka, Marcin Nowotny

**Affiliations:** Laboratory of Protein Structure, International Institute of Molecular and Cell Biology, Warsaw 02-109, Poland

## Abstract

Rad2/XPG belongs to the flap nuclease family and is responsible for a key step of the eukaryotic nucleotide excision DNA repair (NER) pathway. To elucidate the mechanism of DNA binding by Rad2/XPG, we solved crystal structures of the catalytic core of Rad2 in complex with a substrate. Rad2 utilizes three structural modules for recognition of the double-stranded portion of DNA substrate, particularly a Rad2-specific α-helix for binding the cleaved strand. The protein does not specifically recognize the single-stranded portion of the nucleic acid. Our data suggest that in contrast to related enzymes (FEN1 and EXO1), the Rad2 active site may be more accessible, which would create an exit route for substrates without a free 5′ end.

## INTRODUCTION

Nucleotide excision repair (NER) is one of the main DNA repair pathways (1). Its characteristic feature is the ability to detect and remove a wide variety of DNA modifications with various chemical structures. NER begins with the detection of the lesion, which in eukaryotes is performed by a complex of XPC and RAD23B proteins (Rad4 and Rad23 in *Saccharomyces cerevisiae*). Additionally, in higher eukaryotes, a specialized complex called UV-DDB, comprising the DDB1 and DDB2 proteins, specifically recognizes ultraviolet (UV)-induced lesions. A general transcription factor, TFIIH, is then recruited. It is a large complex of 10 subunits, including two helicases of opposite polarity: XPD and XPB. The XPA protein and RPA heterotrimer then bind to form the pre-incision complex, and two nucleases are recruited to perform cuts on each side of the lesion, allowing the removal of the damaged DNA fragment of ∼30 nucleotides (nt). XPG (Rad2 in yeast) is responsible for the 3′ cut, and the XPF–ERCC1 complex (Rad1–Rad10 in yeast) complex, in which the XPF is the catalytic subunit, executes the 5′ cut. After the damaged DNA fragment is removed, DNA repair synthesis occurs.

In humans, mutations in NER components lead to diseases, such as xeroderma pigmentosum (XP) with extreme susceptibility to UV radiation and an increased risk of skin cancer (2), Cockayne syndrome (CS) with developmental impairment, premature aging and sunlight sensitivity, and trichothiodystrophy with developmental impairment and mental retardation (3). XPG was initially discovered as complementation group G of XP and hence was given its name (4). In addition to XP, mutations in XPG can also lead to XP with symptoms of CS (5).

Rad2/XPG belongs to the flap endonuclease family that encompasses various structure-specific nucleases involved in nucleic acid processing (5–7). Their nuclease domain comprises two sequence segments (N and I) that in the three-dimensional structures are highly intertwined and form the catalytic core of the enzyme (8,9). In most family members, the two segments are separated by a relatively short linker, but a unique sequence of ∼600 amino acids termed the ‘spacer region’ is present in Rad2/XPG between the N and I regions (7). It does not resemble any known proteins, most of its sequence is not conserved and it is predicted to be mostly disordered. Spacer region fragments mediate the interactions with other components of NER, including TFIIH and RPA (10–15). The interaction between one of these Rad2 fragments and TFIIH component Tfb1 has been structurally characterized (16). The C-terminal region of Rad2/XPG contains a nuclear localization signal and a PIP motif that interacts with proliferating cell nuclear antigen (PCNA) and is involved in UV-induced mutagenesis (17).

In the pre-incision complex, XPG becomes fully active once the 5′ incision is made by the other NER nuclease: XPF-ERCC1 (18). However, both the isolated Rad2 and XPG proteins efficiently cleave various DNA substrates *in vitro* (19–24). These include DNAs with single-stranded 5′ overhangs (20,22) and splayed-arm DNAs, both of which are cleaved in the strand with the 5′ arm. The unique feature of Rad2/XPG is the recognition of DNA bubbles that comprise a stretch of unpaired bases flanked by double-stranded regions and correspond to the DNA in the NER pre-incision complex. All of the substrates are cleaved in the vicinity of the junction between the single-stranded and double-stranded DNA (ss/dsDNA junction), with the main cleavage site 1 nt into the double-stranded region. Rad2/XPG cleaves both splayed-arm and bubble substrates with similar efficiency (10), suggesting that it processes each of the two ss/dsDNA junctions in the bubble DNA independently. The exact basis of substrate recognition, however, is unknown.

In addition to Rad2/XPG, other prominent members of the flap endonuclease family include FEN1, which is involved in DNA replication, EXO1, which participates in mammalian mismatch repair and double-strand break repair (25,26), and GEN1, one of the eukaryotic Holliday junction resolvases (27,28). The preferred substrate for FEN1 is a nicked duplex with both 5′ and 3′ single-stranded flaps with preference for the 1 nt 3′ flap. Recent crystal structures of FEN1 substrate complexes showed that the two double-stranded regions that flank the flaps are at a right angle when bound by FEN1 (29). The substrate for EXO1 exonuclease is a duplex with a 3′ single-stranded overhang, and the mechanism of its recognition was recently elucidated based on crystal structures (30). Both FEN1 and EXO1 use several critical elements for substrate binding. The first element is the helix-two turn-helix (H2TH) motif that is stabilized by a potassium ion and binds the double-stranded region downstream from the active site. It interacts with the non-cleaved strand approximately one helical turn from the scissile phosphate. The first exposed base pair of the downstream double-stranded region is bound by an element termed the helical wedge, which is made of two α-helices. At the active site, the DNA is clamped by two other α-helices that form an element termed the ‘helical arch’, which is partially disordered in some structures (31–34). Other members of the flap nuclease family for which structural information is available include bacterial nuclease ExoIX (35) and phage T4 RNase H (9,31). Despite the importance of Rad2/XPG for NER and its relevance to human disease, no structural information is available for the catalytic core of this enzyme.

The active sites of flap nucleases are formed by conserved carboxylate residues that coordinate catalytic divalent metal ions. Catalysis is thought to occur through a two-metal ion mechanism (36) that is utilized by many other nucleic acid enzymes (37). In the pre-reactive state, the scissile phosphate does not productively interact with the metal ions, and a conformational change of the DNA is required for hydrolysis. This ‘fraying’ involves the unpairing of one base on each side of the scissile phosphate and its translocation into the active site (29,30). Biochemical experiments that used DNA substrates with blocked DNA ends and single-molecule fluorescence resonance energy transfer (FRET) studies showed that the substrate binding by FEN1 proceeds through a mechanism termed ‘disorder-thread-order’, in which the 5′ flap is initially threaded under a partially unfolded helical arch that then becomes fully ordered and clamps the DNA to allow hydrolysis (38,39).

Because no structural information is available for the catalytic core of Rad2/XPG, and the mechanism of its unique specificity for bubble substrates is unknown, we sought to solve the crystal structures of this protein in complex with DNA substrate. We report four structures of the Rad2 catalytic core solved in four different space groups, three of which represent the productive substrate binding mode and reveal that the protein recognizes a single 5′ nucleotide of the single-stranded portion of the DNA and a 3′ phosphate group of the ss/dsDNA junction. The two critical regions for substrate binding are the H2TH module with a Rad2-specific α-helix 12b and the hydrophobic wedge that binds the first base pair of the double-stranded portion of the DNA. Our structures suggest that the helical arch may adopt a different structure in Rad2/XPG than in FEN1 and EXO1. This alteration of the Rad2 structure forms an opening and creates an exit route from the active site. This may allow for the cleavage of DNA bubbles that do not possess a free DNA 5′ end.

## MATERIALS AND METHODS

### Protein expression and purification

*Rad2* gene was amplified by polymerase chain reaction (PCR) from *S. cerevisiae* genomic DNA and cloned into a pET28–6xHis-SUMO vector using SLIC (sequence- and ligation-independent cloning) (40). Using the same approach, the Rad2 open reading frame with 500 flanking nucleotides was cloned in pRS413 vector. The mutations were introduced in the plasmid using site-directed PCR mutagenesis according to standard protocols. The protein variants, including Sc-Rad2-ΔS (Δ112-731), Sc-Rad2-ΔSC (Δ112-731, Δ987-1031), and their derivatives, were expressed in *Escherichia coli* BL21 (DE3) Rosseta. For protein expression cells were grown in Luria Broth (LB) medium at 37°C, induced with 0.04 mM Isopropyl β-D-1-thiogalactopyranoside (IPTG) at OD_600_ = 0.6–0.9 and grown overnight at 15°C. The cells were harvested by centrifugation, and dry pellets were stored at −20°C. Selenomethionine-labeled Sc-Rad2-ΔSC protein was expressed in SelenoMethionine Expression Media (Molecular Dimensions) using the same protocol.

For protein purification, an appropriate pellet was thawed on ice and further lysed by sonication in buffer that contained 50 mM Tris-HCl (pH 7.5), 500 mM NaCl, 40 mM imidazole, 5% (v/v) glycerol and 5 mM 2-mercaptoethanol (buffer A). The lysate was clarified by centrifugation at 40 000 rotations per minute (rpm), and the supernatant was loaded on a HisTrap column (GE Healthcare) equilibrated in buffer A. The purification procedure included a wash with buffer A that contained 2 M NaCl, a second wash with buffer A with 70 mM imidazole and protein elution with buffer A with 300 mM imidazole. 6xHis-SENP protease was added to the protein-containing fractions, which were then dialyzed overnight in a buffer that contained 20 mM Tris-HCl (pH 7.5), 150 mM NaCl, 5% (v/v) glycerol and 5 mM 2-mercaptoethanol. Dialyzed samples were re-applied on a HisTrap column, and protein that contained flow-through was concentrated on an Amicon Centrifugal Filter Device (Millipore). For crystallization purposes, the protein was further purified on a Superdex 200 size exclusion column (GE Healthcare) in buffer that contained 20 mM HEPES (pH 7.5), 150 mM KCl or NaCl, 5% (v/v) glycerol and 0.5 mM TCEP with or without the addition of 5 mM (CH_3_COO)_2_Ca. Peak enzyme-containing fractions were concentrated on an Amicon device. The protein used for the crystallization of complex I and biochemical studies was stored in buffer that contained 150 mM NaCl. The protein used for all other crystallizations was stored in buffer that contained 150 mM KCl and 5 mM (CH_3_COO)_2_Ca. Selenomethionine-labeled Sc-Rad2-ΔSC protein variant was purified using the same procedure. The protein purification procedures were performed using ice-cold buffers, and purified proteins were stored at 4°C or −80°C.

### Crystallization

High-performance liquid chromatography (HPLC)-purified DNA oligonucleotides were purchased from Metabion International AG. Prior to the crystallization experiments, protein was mixed with DNA at a 1:1.2 molar ratio, with a final Rad2-ΔSC protein concentration of 10 mg/ml. The oligonucleotides used for crystallization comprised a 12–15 bp duplex region and 1–4 nt overhangs in one or both ends of the double-stranded part (for the sequences, see Table 1). All of the experiments were performed at room temperature using a sitting-drop vapor diffusion approach.

**Table 1.**
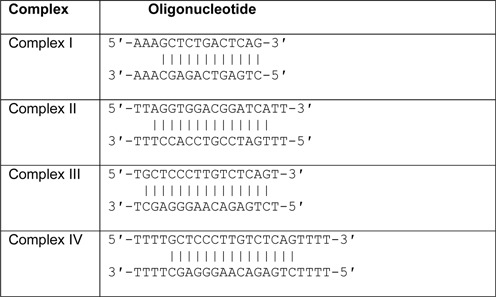
Sequences of oligonucleotides for crystallization

The initial crystallization condition for complex I was identified using Crystal Screen (Hampton Research). After optimization, the best diffracting crystals were obtained by mixing the protein–DNA complex with an equal volume of reservoir buffer that contained 20% (v/v) ethylene glycol. Before data collection, the crystals were cryoprotected by the addition of 50% (v/v) glycerol to a final concentration of 25% (v/v) and flash frozen in liquid N_2_. For the crystallization of selenomethionine-labeled protein, an additional 1 mM TCEP concentration was used.

The crystals of complexes II, III and IV were initially obtained from Morpheus screen (Molecular Dimensions). The best diffracting crystals of complex III were obtained by mixing protein–DNA complex with an equal volume of reservoir buffer that contained 9% (w/v) PEG 20000, 20% (v/v) PEG MME 550, 0.02 M D-glucose, 0.02 M D-mannose, 0.02 M D-galactose, 0.02 M L-fructose, 0.02 M D-xylose, 0.02 M *N*-acetyl-D-glucosamine and 0.1 M MOPS/HEPES-Na, pH 7.5. The crystals were cryoprotected by the addition of 50% (v/v) PEG MME 550 to a final concentration of 25% (v/v) and flash frozen in liquid N_2_.

The crystals of complex III were obtained by mixing protein–DNA complex with an equal volume of reservoir buffer that contained 10% (w/v) PEG 8000, 20% (v/v) ethylene glycol, 0.02 M D-glucose, 0.02 M D-mannose, 0.02 M D-galactose, 0.02 M L-fructose, 0.02 M D-xylose, 0.02 M *N*-acetyl-D-glucosamine and 0.1 M MES/imidazole, pH 6.5. The crystals were cryoprotected by the addition of 50% (v/v) MPD to a final concentration of 25% (v/v) and flash frozen in liquid N_2_.

The crystals of complex IV were grown by mixing protein–DNA complex with an equal volume of reservoir buffer that contained 8% (w/v) PEG 8000, 18% (v/v) ethylene glycol, 0.02 M D-glucose, 0.02 M D-mannose, 0.02 M D-galactose, 0.02 M L-fructose, 0.02 M D-xylose, 0.02 M *N*-acetyl-D-glucosamine and 0.1 M MOPS/HEPES-Na, pH 7.5, and flash frozen in liquid N_2_.

### Diffraction data collection, structure solution and refinement

X-ray diffraction data were collected at 14.1 and 14.2 beam lines at Berliner Elektronenspeicherring-Gesellschaft für Synchrotronstrahlung (BESSY) (41). Diffraction data were processed and scaled with HKL2000 (42). The statistics of the diffraction data are summarized in Table 2. The complex I structure was solved using single-wavelength anomalous diffraction in AutoSol module in Phenix (43) using data collected at selenium peak wavelength (0.97989 Å). Diffraction data for complexes II, III and IV were collected at 0.89440 Å wavelength and the structures were solved by molecular replacement in PHASER (44) using the protein model from complex I as a search model. Interactive model building was performed in COOT (45) and refinement with Phenix (43) with R-free, calculated with 5% of unique reflections. According to Molprobity analysis (46), in the final models, the following percentages of residues were located in the allowed regions of the Ramachandran plot: complex I - 99.1%, complex II - 100%, complex III - 99.9% and complex IV - 99.8%. Structural analyses, including superpositions, and structural figures were prepared in Pymol (http://www.pymol.org).

**Table 2.** Data collection and refinement statistics

	Complex I (SeMet)	Complex II	Complex III	Complex IV
**Data collection**				
Space group	*P*3_2_21	*P*2_1_2_1_2	*P*2_1_	*C*2
Cell dimensions				
*a*, *b*, *c* (Å)	94.08, 94.08, 155.26	126.64, 76.81, 108.19	110.28, 86.04, 134.44	98.68, 97.96, 112.04
α, β, γ (°)	90, 90, 120	90, 90, 90	90, 110.95, 90	90, 96.41, 90
Resolution (Å)	40.0–2.75 (2.8–2.75)	40.0–2.1 (2.14–2.1)	40.0–2.4 (2.44–2.4)	50.0–2.7 (2.75–2.7)
*R*_merge_ (%)	11.4 (46.6)	8.8 (83.1)	11.4 (75.1)	7.1 (60.3)
*I* / σ*I*	29.9 (2.2)	25.2 (2.1)	14.3 (1.9)	19.7 (1.9)
Completeness (%)	98.8 (86.5)	99.8 (99.3)	100.0 (99.5)	95.8 (99.9)
Redundancy	10 (5.5)	7.1 (6.3)	4.0 (3.9)	3.4 (3.3)
				
**Refinement**				
Resolution (Å)	2.75	2.1	2.4	2.7
No. reflections	39066	62159	92066	27918
*R*_work_/*R*_free_	24.53/31.02	18.34/22.99	17.80/23.55	23.16/28.41
No. atoms	4556	6154	13433	6126
Protein	4395	4967	9942	4670
DNA	82	616	2660	1326
Water	79	567	823	126
Ion	-	4	8	4
Average*B*-factors (Å^2^)	52.6	32.0	33.2	66.3
Protein	52.5	30.1	31.1	60.5
DNA	61.7	42.2	38.0	88.0
Ion	-	53.6	58.0	60.7
Water	45.8	37.3	34.9	55.0
Root-mean-square deviations				
Bond lengths (Å)	0.090	0.007	0.008	0.007
Bond angles (°)	1.163	1.053	1.123	1.006

Values in the parentheses are for the highest resolution shell.

### Rad2 cleavage assay

The HPLC-purified DNA oligonucleotides (Metabion International AG) listed in Supplementary Table S1 were used to create the splayed-arm (Y1, Y2) and bubble (B1, B2) DNA substrates. Appropriate oligonucleotides were labeled using [γ-^33^P]-adenosine 5′-triphosphate (Hartmann Analytic GmbH) and T4 Polynucleotide Kinase (Thermo Scientific) according to the manufacturer's protocols and annealed with a 2-fold excess of unlabeled complementary oligonucleotides. Incision assays on Rad2-ΔSC protein and its variants were performed in buffer that contained 25 mM Tris (pH 7.5), 30 mM KCl, 2.5 mM 2-mercaptoethanol and 0.5 mM MnCl_2_ at 37°C with 90 min reaction time using 2 nM substrate and 5, 10, 20, 40 and 80 nM enzyme. The experiments that compared the activity of Rad2-ΔSC and Rad2-ΔS variants were performed in the same buffer as above but at 30°C with 90 min incubation using 2.5 nM substrate and 0.2, 0.5, 1.0, 2.0, 3.5, 5.0, 10.0, 20.0 and 40.0 nM enzyme. The reactions were stopped by the addition of an equal volume of formamide loading buffer (90% [v/v] formamide and 0.1% [w/v] bromophenol blue) and heating for 10 min at 95°C. The samples were loaded onto 20% denaturing TBE-urea (Tris-Borate-EDTA) ( (7 M) polyacrylamide gel (19:1 cross-linking ratio) that contained 1x TBE and run for 1.5–2 h at 20 W. The experiments were visualized by autoradiography using Typhoon Trio+ (GE Healthcare) and quantification was performed in ImageQuant TL 7.0 software.

### UV sensitivity complementation

*Rad2*Δ strain (Y07289: BY4741; MAT **a**; *his3*Δ1; *leu2*Δ0; *met15*Δ0; *ura3*Δ0; YGR258c::kanMX4) was obtained from EuroScarf (http://web.uni-frankfurt.de/fb15/mikro/euroscarf/index.html). It was transformed using lithium acetate method with the empty pRS413 vector or its counterparts with the wild-type *RAD*2 ORF (Open Reading Frame) with flaking 500 nt and its point substitution variants. The transformants were cultured in liquid selective medium for 2–3 days. Serial dilutions of these cultures were plated on YPD (Yeast extract Peptone Dextrose) or minimal media plates. After drying, the plates were exposed to UV radiation (10–100 J/m^2^) using CL1000 UV-crosslinker (UVP, LLC, Upland, CA) and were incubated for 2–3 days at 28°C.

## RESULTS

### Structure solution

To obtain structural information on the Rad2 protein, we first produced the full-length fungal orthologs. They underwent extensive crystallization trials but did not produce crystals. We therefore decided to work with deletion mutants. The central spacer region of Rad2/XPG is predicted to be mostly disordered. Its deletion in *S. cerevisiae* Rad2 resulted in active protein (6) and an XPG variant with spacer residues 111–730 removed showed wild-type protein levels of activity on splayed-arm substrates and ∼40% activity on bubble substrates (10). We prepared a variant of *S. cerevisiae* Rad2, corresponding to this XPG deletion mutant (residues 112–731 were removed) and we termed it Sc-Rad2-ΔS (Supplementary Figure S1). The activity of Sc-Rad2-ΔS could not be directly compared with the full-length Sc-Rad2 because this protein, although expressed in *E. coli*, was unstable during purification, prone to degradation and aggregation and unsuitable for activity assays. However, we did compare the activity of our deletion variants with the published data for full-length XPG (10). Under the same conditions the activity of both XPG and Sc-Rad2-ΔS reached a plateau at ∼80% of the substrate cleavage [(10) and Supplementary Figure S2]. At the same substrate concentration, 0.25 nM XPG and 3 nM Rad2-ΔS were required to achieve half of the maximal substrate cleavage. Therefore, the activity of Sc-Rad2-ΔS corresponded to ∼8% of the activity of the full-length XPG, which may also be a result of differences between the human and yeast enzyme. Importantly, XPG and Sc-Rad2-ΔS cleaved splayed-arm and bubble substrates with similar efficiency (Supplementary Figure S2) demonstrating that the deletion of the spacer region did not affect the substrate specificity of Rad2.

In the second deletion mutant, Sc-Rad-ΔSC, we also removed the C-terminus (residues 987–1031) that contains the nuclear localization signal and PCNA-binding PIP-box motif. This was motivated by the fact that these motifs are most likely disordered and a FEN1 variant with a very similar deletion has been successfully used for structural studies (29). The activity of this FEN1 variant was reduced 5-fold in comparison with full-length protein which was attributed to reduced substrate affinity that results from the loss of nonspecific interactions between the highly positively charged C-terminus of FEN1 and DNA (29). For Sc-Rad2-ΔSC, the activity was difficult to compare with XPG, because its activity plateaued at a much lower percentage of substrate cleavage. However, similar to FEN1, the removal of the C-terminus in Rad2-ΔSC also reduced the activity (Supplementary Figure S2). Nonetheless, Sc-Rad-ΔSC retained enzymatic activity and substrate specificity and comprises the catalytic core of the enzyme. Therefore, it is a good model for structural studies.

**Figure 1.**
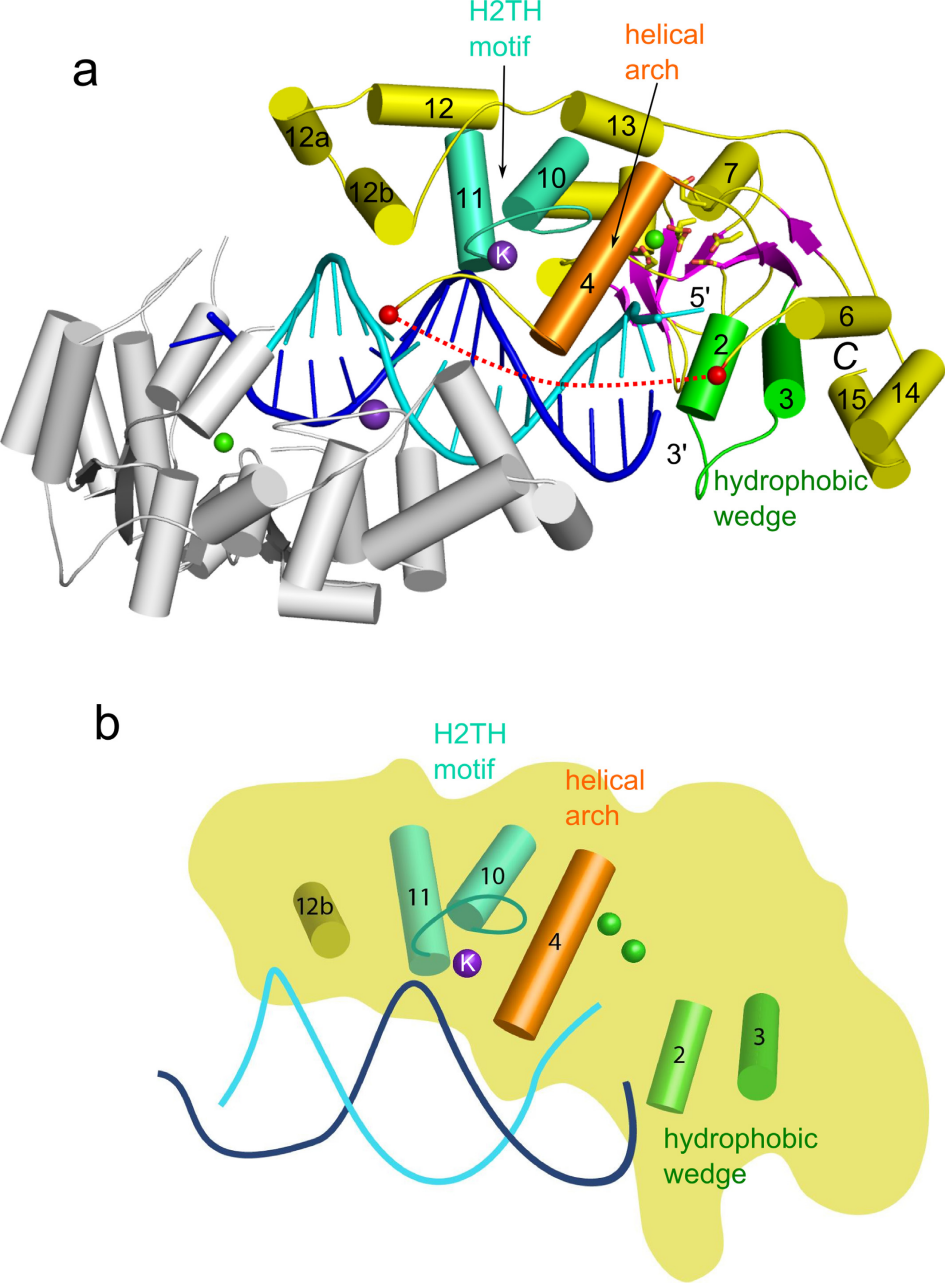
Overall structure of Sc-Rad2-ΔSC in complex with DNA (complex II). (**a**) One protein subunit of the complex is shown in gray, and the other is shown in color (green for hydrophobic wedge, orange for helical arch, green cyan for H2TH motif, magenta for β-strands and yellow for the rest of the structure). The active-site residues are shown as sticks, and calcium and potassium ions are shown as green and purple spheres, respectively. The DNA strand located in the vicinity of the active site of the subunit in color is shown in cyan, and the other strand is shown in blue. Helices are labeled with numbers as in Ref. (29). Red spheres and dashed line mark the region of the insertion of the spacer domain in full-length protein. (**b**) Cartoon representation of the key functional elements of the structure labeled as in (a).

Sc-Rad-ΔSC underwent extensive crystallization trials alone and in the presence of oligonucleotides with a 12–15 bp double-stranded region and single-stranded overhangs made of one to six adenines or thymines (DNA sequences are given in Table 1). We did not obtain crystals of the protein alone but we solved four crystal structures of complexes between Rad2-ΔSC and DNA, which we termed complexes I–IV and which are described in detail in the Supplementary Information. Briefly, complex I comprises a Rad2-ΔSC dimer that interacts with disordered DNA. Although it may represent a functional state of the enzyme, we assume it is rather a non-productive complex (Supplementary Figure S3). Complex II was solved at 2.1 Å resolution and comprised two protein molecules, each interacting with one ss/dsDNA junction (Figure 1). The substrate used for crystallization contained 14 complementary bases with two-thymine single-stranded overhangs in all four DNA ends. However, in the crystal a single T-T mismatch formed, resulting in a 15-mer duplex with 1 nt overhangs in one end of the duplex and 2 nt overhangs in the other. The structure of complex III was solved at 2.4 Å resolution. It comprised two Rad2-ΔSC molecules bound to a 15-mer DNA duplex with 1 nt single-stranded overhangs in all four ends of the DNA strands. Two copies of complex III were present in the asymmetric unit of its crystals. Complex IV was solved at 2.7 Å resolution and comprised two protein molecules and a 15-mer DNA substrate with 4 nt single-stranded overhangs in all four ends of the DNA strands. The productive complexes II–IV have the same overall architecture. The relative orientation of the protein molecules is similar between complexes II and III but differs in complex IV (Supplementary Figure S4a). These differences are attributable to crystal packing and the flexibility of the DNA.

All eight independently determined protein structures in complexes II–IV are essentially identical (Supplementary Figure S4b), with pairwise root-mean-square deviations (rmsds) between 0.5 and 0.8 Å over 273 to 285 C-α atoms. As expected, the catalytic core of Rad2 is quite similar to the other members of the flap nuclease family (29–31,35). Its molecule has an oblong shape with the active site located roughly in the middle. The central element of the structure is a twisted central β-sheet made of seven strands flanked by α-helices (Figure 1). For ease of comparisons, we adopted the same numbering scheme for secondary structure elements as in the previous work on FEN1 and EXO1 (29). Additional helices observed in Rad2 are designated α12a and α12b (Figure 1 and Supplementary Figure S1).

### Substrate binding

When all of the protein subunits from the productive complexes II–IV are compared, the DNA superimposes very well at the H2TH module, but its trajectory differs significantly in other regions, particularly around the hydrophobic wedge (Supplementary Figure S5). This indicates the flexibility of DNA binding and mobility of the substrate, which may be important for the DNA ‘fraying’ and cleavage.

**Figure 2.**
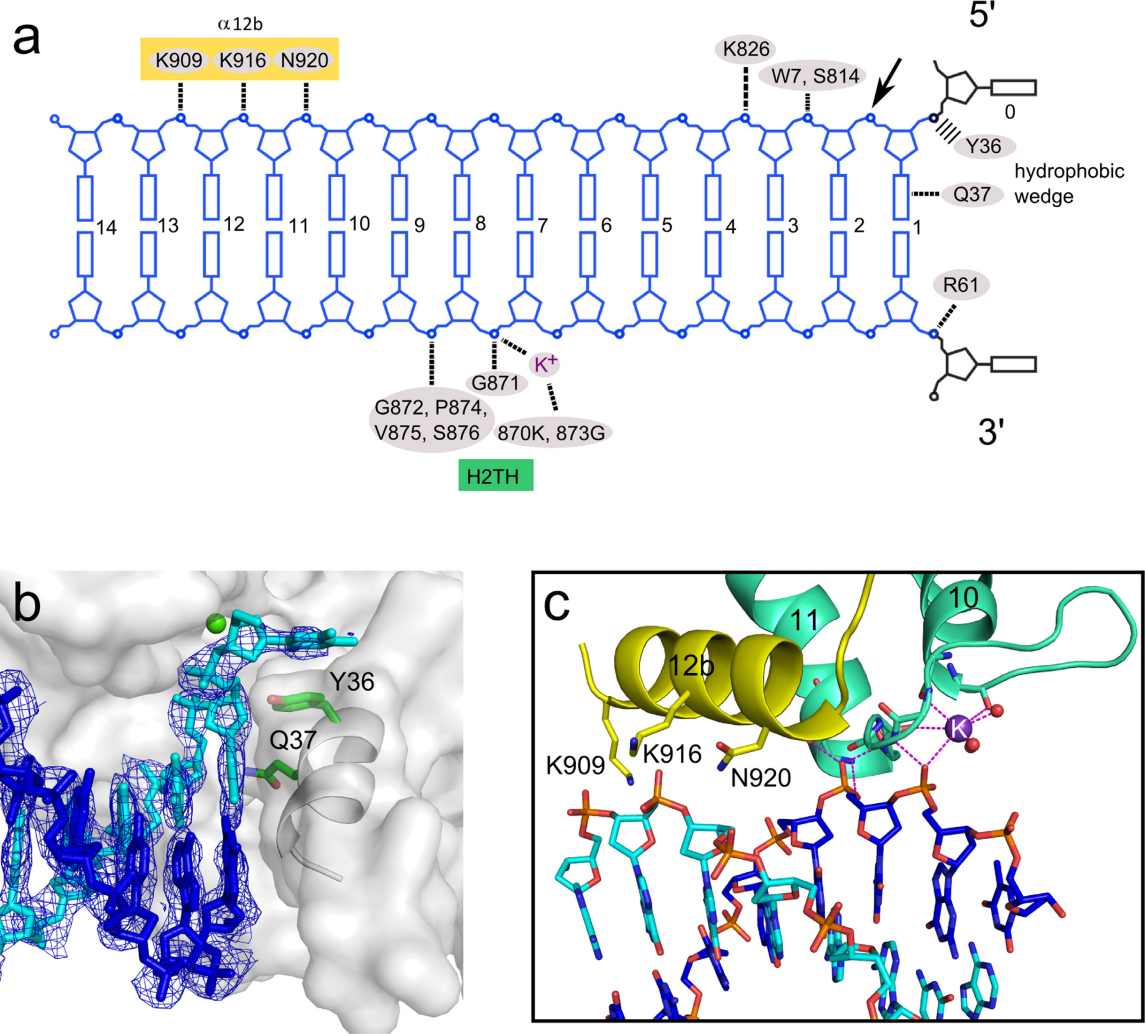
Substrate binding. (**a**) Diagram of protein–DNA interactions. The preferred site of cleavage is indicated with an arrow. (**b**) Interactions with the hydrophobic wedge. The protein is shown in transparent surface representation, with helix α2 shown in a gray cartoon. The substrate is shown in cyan for the cleaved strand and blue for the non-cleaved strand. Calcium ion at the active site is shown as a green sphere, and the two residues involved in substrate binding are shown as green sticks. A 2Fo-Fc simulated annealing composite omit map contoured at 1.2 σ is overlaid on the DNA. (**c**) H2TH motif. The DNA is colored as in (b). The potassium ion is shown as a purple sphere.

In our structures, including complex IV in which the DNA had the longest overhangs (4 nt), we did not observe electron densities for the single-stranded regions of the DNA, except for the single nucleotide in the cleaved (5′ flap) strand that is visible in complexes III and IV. The phosphate group on the 3′ side of this single nucleotide is stabilized by van der Waals interactions with Tyr36 (from N region) (Figure 2a and b). For the non-cleaved (3′ flap) DNA strand, in one protein molecule of complex II and three protein molecules of complex III, we observed the electron density for the phosphate group of the ss/dsDNA junction. Several charged residues line a pocket located close to this phosphate: Arg60, Arg61 (both from the N region), His830 and Glu831 (from I region) (Supplementary Figure S6a and b). Arg61 is absolutely conserved among Rad2/XPG proteins, and Arg60 is replaced by a histidine in many sequences. Residues His830 and Glu831 are less conserved but nearly always replaced by polar amino acids. In complex II, Arg60 and potentially Arg61 (through a water molecule) stabilize the phosphate at the junction.

A single Sc-Rad2-ΔSC protein molecule covers 13 bp of the double-stranded portion of the substrate, with two regions of protein–DNA interactions (Figure 2a). The first comprises the contacts with the ss/dsDNA junction and interactions around the active site. The second region includes the H2TH domain and Rad2-specific helix α12b. The first region contains a protrusion formed by the ‘hydrophobic wedge’ made of α-helices 2 and 3 and located in the vicinity of the active site (Figure 2b). The first exposed base pair of the DNA abuts this protrusion. Extensive van der Waals interactions are formed between the aromatic rings of the first base pair and helix 2 (Figure 2b). Gln37 from helix 3 (N region), conserved in nearly all Rad2/XPG proteins, is located close to the edge of the first base pair and in several complexes forms a hydrogen bond with the base of the cleaved strand. Protein–DNA interactions mediated by the hydrophobic wedge are the only ones that involve the bases; all other contacts occur through the phosphodiester backbone of the substrate.

The Rad2 active site is made of Asp30, Asp77 (both from N region), Glu792, Glu794, Asp813, Asp815 and Asp864 (all from I region) (Supplementary Figure S7). The architecture of the Rad2 active site is nearly identical to those of FEN1 and EXO1. Calcium ions were present in the crystallization conditions, and we observed electron density for one metal ion at the active sites. None of phosphate groups of the DNA interacts with the active site in a manner conducive for cleavage. This implies that, similar to FEN1 and EXO1, substrate ‘fraying’ is required for hydrolysis. Downstream from the active site, the cleaved strand of the DNA forms interactions with Trp7 and Lys826 (Supplementary Figure S6c). The middle portion of the DNA (nt +4 to +7; throughout the text, DNA base pairs are numbered from the ss/dsDNA junction) does not interact with the protein and is exposed to the solvent. Nucleotides +8, +9 and +10 of the non-cleaved strand are tightly bound by the H2TH module located in the I region and stabilized by a potassium ion (Figure 2c). The ion is coordinated by backbone carbonyls of Leu869 and Met872 and three water molecules and interacts with the phosphate of nt +8. Phosphates of nt +8 and +9 form hydrogen bonds with the backbone of Gly871, Gly873, Val875, Ser876 and the side chain of Ser876 (Figure 2a). Further downstream, three charged residues (Asn920, Lys916 and Lys909), all located on one face of helix α12b, form a dynamic network of interactions with the cleaved strand phosphates of nt +9, +10 and +11. In each complex structure, we determined this network is slightly rearranged because of the change in the trajectory of the DNA. The sequence region that corresponds to helix α12b exhibits little conservation at the level of amino acid sequence among Rad2/XPG proteins, but in numerous orthologos from plants, fungi and higher eukaryotes it comprises polar residues and forms an α-helix according to Genesilico Metaserver (47) secondary structure prediction. We speculate that the Rad2-specific contacts mediated by helix α12b, together with base interactions mediated by Gln37, may compensate for the lack of specific binding of the single-stranded overhangs by Rad2.

In summary, our structures imply that the key interactions with the DNA are mediated by the H2TH module. We do not observe the specific binding of the single-stranded portion of the DNA, and the main substrate specificity determinant is the recognition of the exposed base pair at the ss/dsDNA junction by the hydrophobic wedge.

### Biochemical studies

**Figure 3.**
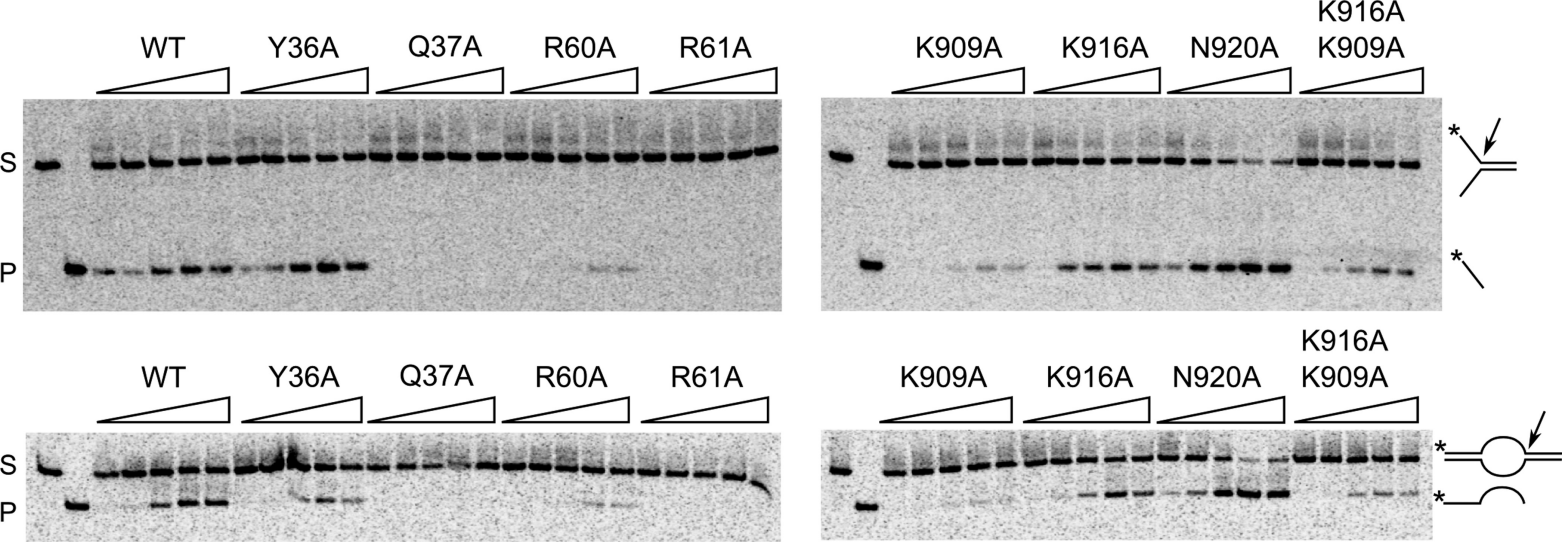
Activity assay for Sc-Rad2-ΔSC variants with point substitutions. Splayed-arm (top panels) and bubble (bottom panels) DNA substrates (2 nM) radiolabeled in the 5′ end were mixed with Rad2-ΔSC point mutants indicated above each gel at increasing concentrations (triangle: 5, 10, 20, 40 and 80 nM). The reactions were performed in the presence of 0.5 mM MnCl_2_ and incubated at 37°C for 90 min. The products of the reaction were visualized by autoradiography. The position of the substrate (S) and product (P) markers is shown in the two leftmost lanes of each gel. Arrows point to the enzyme cleavage site, and the asterisk indicates the position of the radiolabel.

To gain further insights into the mechanism of Rad2, we performed biochemical experiments. We first prepared Sc-Rad2-ΔSC variants with alanine substitutions of residues that were identified in our structures to be important for DNA binding, and we tested their activity on splayed-arm and bubble substrates in the presence of Mn^2+^ ions (Figure 3 and Supplementary Figure S8). For each of the tested variants, the effect was similar for the two DNA substrates. This result indicates that both substrates are processed in the same manner, also validating that splayed-arm DNA is a good model for bubble DNA in crystallographic experiments. The Y36A mutant had wild-type levels of activity, implying that the stabilization of the 5′ phosphate of the ss/dsDNA junction is not a critical feature for Rad2 and that this residue is not essential for substrate ‘fraying’. This is further supported by the fact that Tyr36 is not conserved among different Rad2/XPG sequences and is often replaced by Asn, Val or His. In contrast, the Q37A variant showed only traces of activity (very small amounts of the product could be observed, when a large amount of radioactive signal was used), showing that the hydrogen bond formed by Gln37 with the base at the ss/dsDNA junction (i.e. a specific feature of substrate binding by Rad2) is important for activity.

We also tested the importance of the residues that line the pocket stabilizing the 3′ phosphate of the ss/dsDNA junction (Figure 3 and Supplementary Figure S6a and b). The R61A variant had severely reduced activity (only traces of the product could be observed even with large amounts of radioactivity), while the R60A variant showed approximately half of the activity of the wild-type protein, supporting the importance of binding the 3′ phosphate at the ss/dsDNA junction. Furthermore, we wished to assess the importance of the Rad2-specific contacts mediated by helix α12b. K916A behaved similar to wild-type protein and both the K909A and K909A/K916A variants had reduced activity. Interestingly, the N920A variant was hyperactive. The basis of this effect is currently unclear. The fact that substitutions in helix α12b affect activity confirms their role in substrate binding and the complex effect of these substitutions is consistent with the fact that the network of interactions in this part of the DNA interface is dynamic and flexible. In summary, the *in vitro* activity of Sc-Rad2-ΔSC variants corroborated the findings from the crystal structures.

We next wished to study the effect of the same substitutions *in vivo.* We prepared a complementation vector that contained the *RAD2* gene. This vector and its variants coding for Rad2 with amino acid substitutions were introduced to a *rad2*Δ strain of *S. cerevisiae*. The transformants were irradiated with UV light to monitor their UV sensitivity. The results showed that the wild-type Rad2 and all the point substitution variants were able to efficiently complement UV sensitivity (Supplementary Figure S9). Therefore, even the residual activity of some of our Rad2 variants was sufficient to promote NER.

### Mapping of XPG mutations

Our crystal structures of the catalytic core of Rad2 help explain the effect of many of the mutations that have been described for XP and XP/CS patients. Table 3 lists the described point or insertion mutations and the proposed effect they may have. Many of the observed mutations are likely to disrupt the hydrophobic core of the enzyme and several others may perturb the function of the helical wedge. Supplementary Figure S10 shows the conservation of mutated residues between XPG and Rad2 and the location of Rad2 equivalents in the structure of Sc-Rad2-ΔSC. Out of 10 mutated residues, three are not conserved. One of these residues is L778 in XPG which is mutated to proline. Its equivalent in Rad2 is located in an α-helix and the mutation to proline would disrupt the helical structure. Another non-conserved residue, A874 in XPG, is located in a region important for the structure of the H2TH motif. An interesting mutation is an insertion of 44 amino acids after position 917 (48), which is located in the C-terminus of α12b and would lead to the disruption of Rad2-specific DNA interactions mediated by this helix.

**Table 3.** XPG mutations observed in patients

Mutation	Ref	*S. cerevisiae* equivalent	Location	Postulated effect
A28N	(52)	A28	Central β-sheet	Destabilization of the hydrophobic core of the enzyme
L65P	(53)	L65	α3	Destabilization of the helical wedge
P72H	(54)	P72	Central β-sheet	Destabilization of the hydrophobic core of the enzyme
L778P	(55)	R781	α6	Unclear, likely destabilization of the helical wedge
A792V	(49)	A795	α7, vicinity of the active site	Next to the active site, may lead to subtle structural alterations that affect the sensitive geometry of the catalytic center
G805R	(55)	G808	Between α7 and α8	Destabilization of the hydrophobic core of the enzyme
W814S	(55)	F817	α8	Destabilization of the hydrophobic core of the enzyme
L858P	(48)	L861	*N*-term of α10	Destabilization of the H2TH motif, backbone carbonyl of L861 interacts with a water molecule that coordinates the potassium ion
A874T	(56)	S877	α11	Destabilization of the H2TH motif, disruption of the structure of α11
W968C	(52)	W970	Between α14 and α15	Destabilization of the helical wedge
Insertion of 44 amino acids at position 917	(48)	Position 919	C-terminus of α12b	Disruption of Rad2-specific DNA interactions mediated by α12b

### Comparison with FEN1 and EXO1

**Figure 4.**
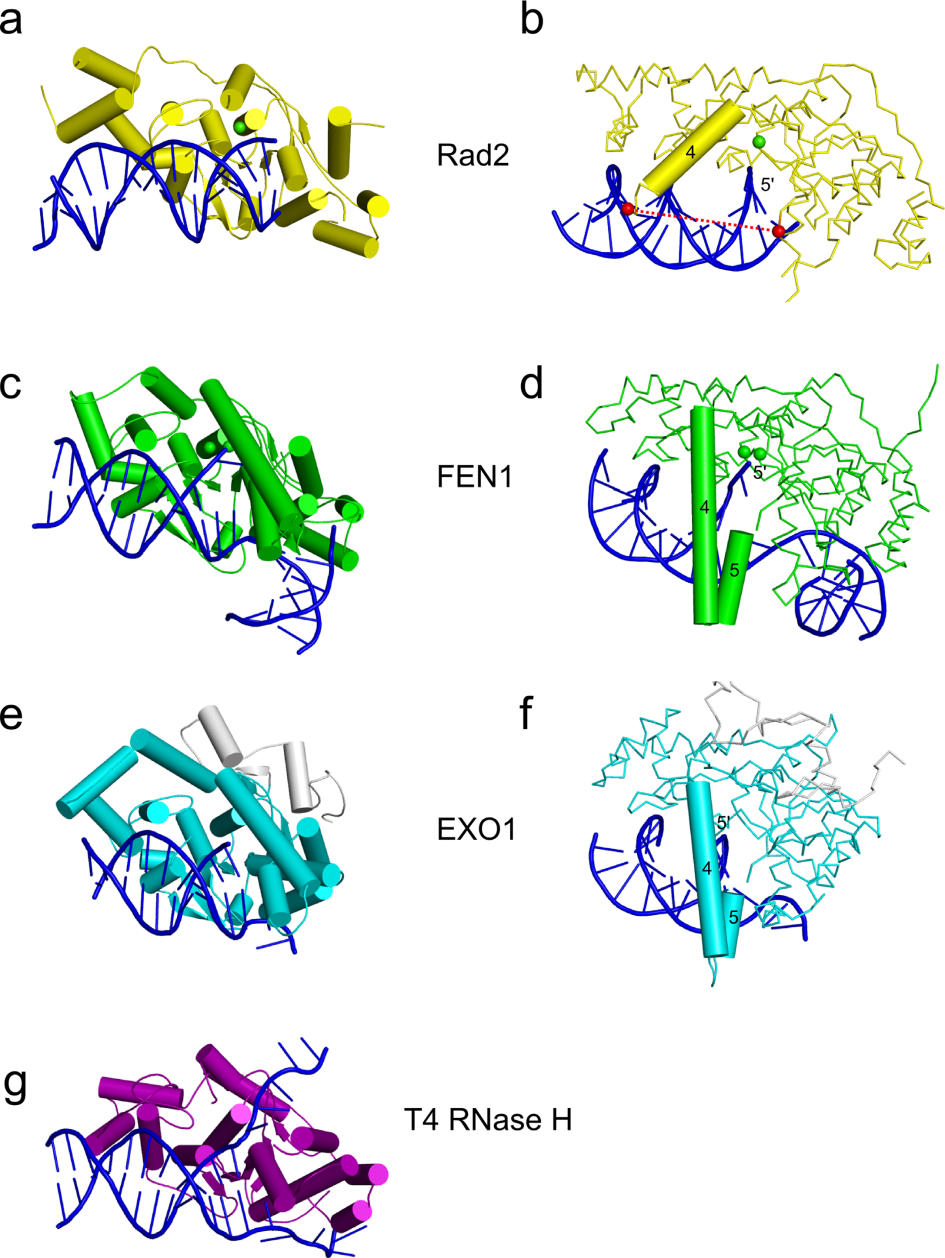
Comparison of Rad2 with other flap nucleases. (**a, b**) Two views of Sc-Rad2-ΔSC complex II. In (b), the C-α backbone trace is shown for the protein, and a cartoon is shown for the helical arch (helix α4). (**c, d**) Two views of FEN1 substrate complex structure (PDB ID: 3Q8K). In (d), the helical arch is shown in cartoon representation. (**e, f**) Two views of EXO1 substrate complex (PDB ID: 3QEB). The additional C-terminal region that is not conserved in other family members is shown in gray. (**g**) Structure of T4 RNase H in complex with DNA substrate.

The structures of Sc-Rad2-ΔSC, FEN1 [e.g. Protein Data Bank (PDB) ID: 3Q8K (29)] and EXO1 [e.g. PDB ID: 3QEA (30)] are similar (Figure 4). The pair-wise superpositions of the catalytic core of Rad2 with EXO1 and FEN1 result in an rmsd of 1.8 Å (207 pairs of C-α atoms) and 2.6 Å (164 pairs of C-α atoms), respectively. When the catalytic cores of the three enzymes are compared, the most conserved elements are the H2TH module and active site (Supplementary Figure S7). Several elements involved in substrate binding are different in Rad2 compared with FEN1 and EXO1. In particular, the catalytic core of Rad2 does not specifically recognize the 3′ overhangs, whereas FEN1 forms extensive and specific contacts with the double-stranded region upstream from the 5′ overhang, and EXO1 binds the single-stranded 3′ overhang. An important element of substrate recognition by Rad2 is Gln37, which forms a hydrogen bond with the base of the cleaved strand. This residue is also conserved in FEN1 but located close to the 3′ phosphate group of the ss/dsDNA junction. In EXO1, Gln37 is conservatively replaced by a lysine, which, however, does not bind the DNA. Therefore, the base recognition by Gln37 is a specific feature of Rad2.

Tyr36 in Rad2 forms a stacking interaction with the base of the single-stranded overhang. Its equivalent in human FEN1, Tyr40, also forms a stacking interaction with the base of nt +2 and participates in DNA ‘fraying’ and the translocation of the scissile phosphate to the active site. Replacement of Tyr40 with alanine reduced the reaction rate by 20-fold, demonstrating the importance of this residue for FEN1 activity (29). Our biochemical experiments showed that Tyr36 is not essential for the activity of Rad2 (Figure 3), so its role is different than in FEN1. Another unique feature of Rad2 is a longer helix α12 (residues 889–899), followed by two additional helices, α12a and α12b, the latter of which (residues 909–921) forms Rad2-specific interactions with the DNA described above.

Another difference that is apparent when Sc-Rad2-ΔSC is compared with FEN1 and EXO1 is the structure of the helical arch. In FEN1 and EXO1, the helical arch comprises two helices, α4 and α5, with the latter playing a critical role as a determinant of substrate specificity by covering the active site and preventing the binding of substrates without free 5′ ends. For Sc-Rad2-ΔSC in all 10 independent determinations of the structure we report herein (including the non-catalytic dimer), helix α5 is missing. It may be a Rad2-specific feature but we cannot exclude a possibility that α5 does not form because of the deletion of the spacer region in the protein variant we crystallized. In Rad2-ΔSC helix α4 adopts a trajectory that is very different from that in FEN1 and EXO1 (Figure 4b, d, and f and Supplementary Figure S4b). Instead of folding over the active site, it points away from it, opening an exit route for the DNA. Helix α4 exhibits some mobility. The position of its C-terminus varies up to ∼6 Å between our structures. This mobility and altered position relative to FEN1 and EXO1 is at least partially attributable to the lack of α5 that stabilizes the conformation of α4 in the other two enzymes.

## DISCUSSION

In this work, we report four substrate complex structures of the Rad2 catalytic core determined with four DNA substrates and in four different space groups. Three of them represent a productive protein–DNA complex, with eight independent determinations of the protein–DNA interactions. Among them, the interaction of the DNA backbone with the H2TH motif is quite invariant, but other regions of the DNA vary in position and trajectory. The mobility of the substrate may result from the lack of interactions that stabilize the single-stranded overhangs. Rad2/XPG normally functions in the incision complex that comprises, among others, TFIIH and XPF. Within this complex, the DNA has to be stabilized to prevent rehybridization of the DNA bubble. Therefore, the bubble structure is pre-formed, and Rad2 does not have to recognize the entire structure. Specific binding of the ss/dsDNA junction may be sufficient to achieve the desired activity.

The lack of single-stranded overhang binding is consistent with biochemical studies. A thorough characterization of the substrate requirements of XPG was performed by Hohl *et al.* (22). Exo III footprinting showed that both the cleaved and non-cleaved strands are bound predominantly in the double-stranded region. In phosphate ethylation interference footprinting, three main regions were protected on the cleaved strand: the 5′ single-stranded overhang 1–4 nt from the junction and regions 2–5 nt and 10–13 nt into the double-stranded region. The latter two regions correspond very well with our structures, particularly with regard to the protection of nucleotides 10–13 by Rad2-specific contacts made by helix α12b. On the non-cleaved strand, only the double-stranded region and not the single-stranded overhang was protected, consistent with our structural data. In the same study, the elements required for splayed-arm DNA substrate cleavage were examined. The reduction of the length of the 5′ arm from 20 nt to 1 or 0 nt resulted in only a 2-fold reduction of activity. Therefore, the 5′ arm was not absolutely required for cleavage. Truncation of the 3′ overhang from the initial 20 nt produced more severe effects: 4-fold reduction of activity for the 2 nt arm and near complete or complete inhibition for the 1 and 0 nt arms, respectively. Therefore, the footprinting assays showed no protection of the 3′ overhang and partial protection of the 5′ strand, whereas the activity assays demonstrated the importance of the 3′ overhang but not the 5′ overhang. Considering our structural data, we propose the following explanation for these results. We do not observe electron density beyond the first nucleotide of the 5′ overhang. Indeed, the protein does not specifically require the 5′ single-stranded region for binding and cleavage. Similarly, we only observe the 3′ phosphate of the junction, so the 3′ overhang is not specifically bound and is disordered in our structures. This may be why it is not protected in footprinting experiments. The disordered mobile 3′ overhang may be needed for activity to promote the ‘fraying’ of the last base pair for proper positioning of the substrate at the active site.

Based on our structures we designed point substitutions, some of which severely affected the activity of Sc-Rad2-ΔSC *in vitro*, in particular Q37A and R61A (Figure 3). Interestingly, all our Rad2 variants were able to rescue the UV sensitivity of the *Rad2*Δ yeast strain (Supplementary Figure S9). A similar phenomenon has been reported for human XPG variants D77E and E791D, which had only residual *in vitro* nuclease activity in isolation but could promote DNA excision in the context of reconstituted NER complex and could restore UV resistance of XP-G cell lines (49). Apparently, even the residual nuclease activity of these published XPG variants and point-substituted Rad2 proteins we studied was sufficient to promote NER. This can be explained by the fact that Rad2/XPG functions in a large multi-protein complex. Other components of this complex likely position the DNA substrate for cleavage and therefore alleviate the substrate binding defects of Rad2/XPG variants (49). Moreover, the 5′ incision by ERCC1-XPF occurs first and is sufficient to start DNA repair synthesis (18). It was also shown that XPG plays a structural role in NER pre-incission complex since only its presence, but not activity, is required for the 5′ incision to occur (49,50). Therefore, the fact that our Rad2 variants complemented UV sensitivity demonstrated that their structural integrity and interactions with the rest of the NER machinery were maintained.

In light of these findings, it is interesting to note that most of the XPG patient mutations are predicted to cause structural alterations of the protein and affect its overall stability, rather than its interactions with DNA directly (Table 3). Therefore, a more severe disruption of XPG stability and function is required for the disease phenotype to arise.

When Rad2-ΔSC structures are compared with those of FEN1 and EXO1, an interesting difference that is apparent is the structure of the helical arch. In Rad2, the trajectory of helix α4 is altered, and helix α5 is missing. Importantly, the ‘spacer’ region in Sc-Rad2 is located between helices α4 and α5. To crystallize the protein, this region had to be deleted, creating the Sc-Rad2-ΔSC construct. It is possible that the lack of the spacer could destabilize the positioning of helix α4 and affect the formation of α5. However, α4 has a similar location in all 10 protein structure determinations from four different space groups (Supplementary Figure S4b), implying that the conformation we observed is stable. Moreover, the region that corresponds to helix α5 was present in the Sc-Rad-ΔSC that we used for crystallization but was disordered in the structures. We could not observe the electron densities of 29 residues of the construct located between helices α4 and α6 in any of the structures. This flexible region should also provide sufficient freedom for α4 to adopt its native conformation. Lastly, the Sc-Rad2-ΔS and Sc-Rad2-ΔSC deletion variants exhibit the substrate specificity of the full-length XPG (Supplementary Figure S2), further suggesting that the conformation of the helical arch observed in our structures may also be a feature of the intact protein.

The altered helical arch structure in Rad2 could be responsible for the unique ability of this enzyme to cleave DNA bubbles. Such substrates have no free 5′ end, and the single-stranded DNA has to go through the active site and exit from it to form the second double-stranded region. The substrate complex structures of FEN1 and EXO1 have no exit route for the 5′ end of the single-stranded DNA (29,30) other than by passing inside the helical arch between helices α4 and α5, which is unlikely based on biochemical experiments (51). For FEN1, the 5′ flap has been postulated to be threaded under the helical arch, which then fully folds to allow cleavage (38,39), however, the exact structural basis of endonucleolytic cleavage by FEN1 remains to be established. For cleavage of the bubble substrates by Rad2/XPG, the threading of the DNA would not be possible because of the lack of a free 5′ end in the DNA. The different position of α4 from the helical arch and lack of helix α5 create an exit route for the DNA without the free 5′ end. Therefore, the altered conformation of the helical arch is a plausible explanation for the substrate specificity of this nuclease.

## ACCESSION NUMBERS

The structures were deposited in the PDB under the following accession codes: 4Q0R, 4Q0W, 4Q0Z and 4Q10 for complexes I, II, III and IV, respectively.

## SUPPLEMENTARY DATA

Supplementary Data are available at NAR Online including.

SUPPLEMENTARY DATA
