# Supplementary material for: Crystal structure of the catalytic core of Rad2: insights into the mechanism of substrate binding
Source: Nucleic Acids Res. 2014 Aug 12;42(16):10762–75. doi: 10.1093/nar/gku729 (PMC4176360; doi:10.1093/nar/gku729)
Supplement: SUPPLEMENTARY DATA [file supp_42_16_10762__index.html]

Crystal structure of the catalytic core of Rad2: insights into the mechanism of substrate binding — Crystal structure of the catalytic core of Rad2: insights into the mechanism of substrate binding — SUPPLEMENTARY DATA 

# Crystal structure of the catalytic core of Rad2: insights into the mechanism of substrate binding

## SUPPLEMENTARY DATA

**Files in this Data Supplement:**

- SUPPLEMENTARY DATA
