# Supplementary material for: Crystal structure of the catalytic core of Rad2: insights into the mechanism of substrate binding
Source: Nucleic Acids Res. 2014 Aug 12;42(16):10762–75. doi: 10.1093/nar/gku729 (PMC4176360; doi:10.1093/nar/gku729)
Supplement: SUPPLEMENTARY DATA [file supp_gku729_nar-00888-m-2014-File006.pdf]

Crystal structure of the catalytic core of Rad2: insights into the mechanism of substrate binding

Michał Miętus[1], Elżbieta Nowak[1], Marcin Jaciuk[1], Paweł Kustosz[1], Justyna Studnicka[1] and Marcin Nowotny[1,*]

[1]Laboratory of Protein Structure, International Institute of Molecular and Cell Biology, Warsaw, 02-109, Poland

[*]To whom correspondence should be addressed: Tel: +48 22 597 07 17; Fax: +48 22 597 07 15; email: mnowotny@iimcb.gov.pl

SUPPLEMENTARY INFORMATION

The Supplementary Information contains Supplementary Results, Supplementary Table 1, Supplementary Figures 1-10

SUPPLEMENTARY RESULTS

Determination of complex structures

The first crystal form was obtained with splayed-arm DNA substrate composed of 12-mer ds-DNA with 5′ and 3′ three-adenine overhangs on one side of the helix (Table 1). It belonged to the $P3_221$ space group, and the structure was solved by single-wavelength anomalous diffraction using selenomethionine-substituted protein crystals and refined to 2.7 Å resolution (Table 2). We termed the structure complex I (Supplementary Fig. 3). It contained two protein molecules arranged with non-crystallographic two-fold symmetry that interacted with each other through a small patch made of residues Lys832, Asn833, Tyr834, and Glu836. We could also observe a weak electron density for a part of the DNA backbone that interacted with the H2TH module of one protein subunit, but the rest of the substrate was disordered. The dimeric arrangement was not reproduced in the other crystal structures we determined later, and the DNA in complex I is disordered. Although it may represent a

functional state of the enzyme, we rather assume that is a non-productive complex. After the structure was refined, we also recognized that the *N*-terminus of the protein used for crystallization was not properly processed by cleavage with SENP protease, leaving two additional residues. Because the amino group of the *N*-terminal glycine in the FEN family participates in active-site formation, these additional residues could prevent proper binding of the DNA in the vicinity of the active site. Therefore, we prepared a new expression construct that contained the *N*-terminal Gly2 upon protease cleavage and performed another round of crystallization trials.

The first structure suggested that the protein should interact with 12 bp of the DNA, so we performed crystallization trials with DNA oligonucleotides with 14 or 15 complementary double-stranded regions with single-stranded overhangs that ranged in length from 0 to 6 nt. Eighty-two different DNA substrates were tested. From these crystallization trials, we obtained three crystal forms. One belonged to the $P2_12_12$ space group, and the structure was solved by molecular replacement using the protein structure from complex I as a search model and refined to 2.1 Å resolution. We called this structure complex II. It contains two protein molecules, each interacting with one ss/dsDNA junction of the DNA. Although the DNA contained 14 complementary bases (Table 1), a 15-mer duplex formed in the crystal with a single T-T mismatch and 1 nt single-stranded overhangs on one side of the double-stranded region and 2 nt overhangs on the other. Two orientations of the DNA were possible. In each, the mismatch could be located near the hydrophobic wedge of either protein molecule. However, only a single orientation was present or at least largely dominant in the structure. We did not observe electron densities beyond 5′ and 3′ phosphate at the ss/dsDNA junction.

Another crystal form was obtained with oligonucleotides that contained a 15 bp of complementary region with four 1 nt single-stranded overhangs (Table 1). It belonged to space group $P2_1$, and the structure was solved and refined to 2.4 Å resolution. The asymmetric unit of these crystals contains two copies of the protein-nucleic acid complex, which is similar to complex II. Two possible orientations of the DNA sequence were refined, each at 50% occupancy, which corresponded well with the electron density maps. We termed this structure complex III. On the 5′ side of all of the ss/dsDNA junctions, we observed electron density for single thymines, which were stabilized by an interaction with Tyr36 (Fig. 2b). All four 3’ phosphates of the ss/dsDNA junction were well defined, and one 3’-terminal thymine was observed making a sharp turn and inserting into the

minor grove of the DNA. Such positioning was only possible because the overhang contained a single nucleotide and probably does not reflect a natural DNA conformation.

An additional crystal form grew in the presence of a substrate with a 15 bp complementary region and longer, four-thymine single-stranded overhangs in all four ends of the DNA (Table 1). The structure belonged to the *C*2 space group and was refined to 2.7 Å resolution with two orientations of the DNA. We termed it complex IV. Like in the previous structures, we did not observe the 3′ overhang beyond the 3′ phosphate of the ss/dsDNA junction, but we did see electron density of the 5′ single-stranded nucleotide stabilized by the interaction with Tyr36. Importantly, this structure confirmed that longer overhangs are also not stably bound by Rad2, except for the 3′ phosphate and first nucleotide of the 5′ overhang.

Supplementary Table 1. Sequences of oligonucleotides for biochemical experiments.

Name	Oligonucleotide sequence
Y1	5′-TCAAAGTCACGACCTAGACACTGCGAGCTCGAATTCACTGGAGTGACCTC
Y2	5′-GAGGTCACTCCAGTGAATTCGAGCTCGCAGCAATGAGCACATACCTAGT
B1	5′-CCAGTGATCACATACGCTTTGCTATTCCGGTTTTTTTTTTTTTTTTTTTTTTTTTTTTTC<br>CGTGCCACGTTGTATGCCCACGTTGACCG
B2	5′-CGGTCAACGTGGGCATACAACGTGGCACGGTTTTTTTTTTTTTTTTTTTTTTTTTTTTT<br>CCGGAATAGCAAAGCGTATGTGATCACTGG

SUPPLEMENTARY FIGURES

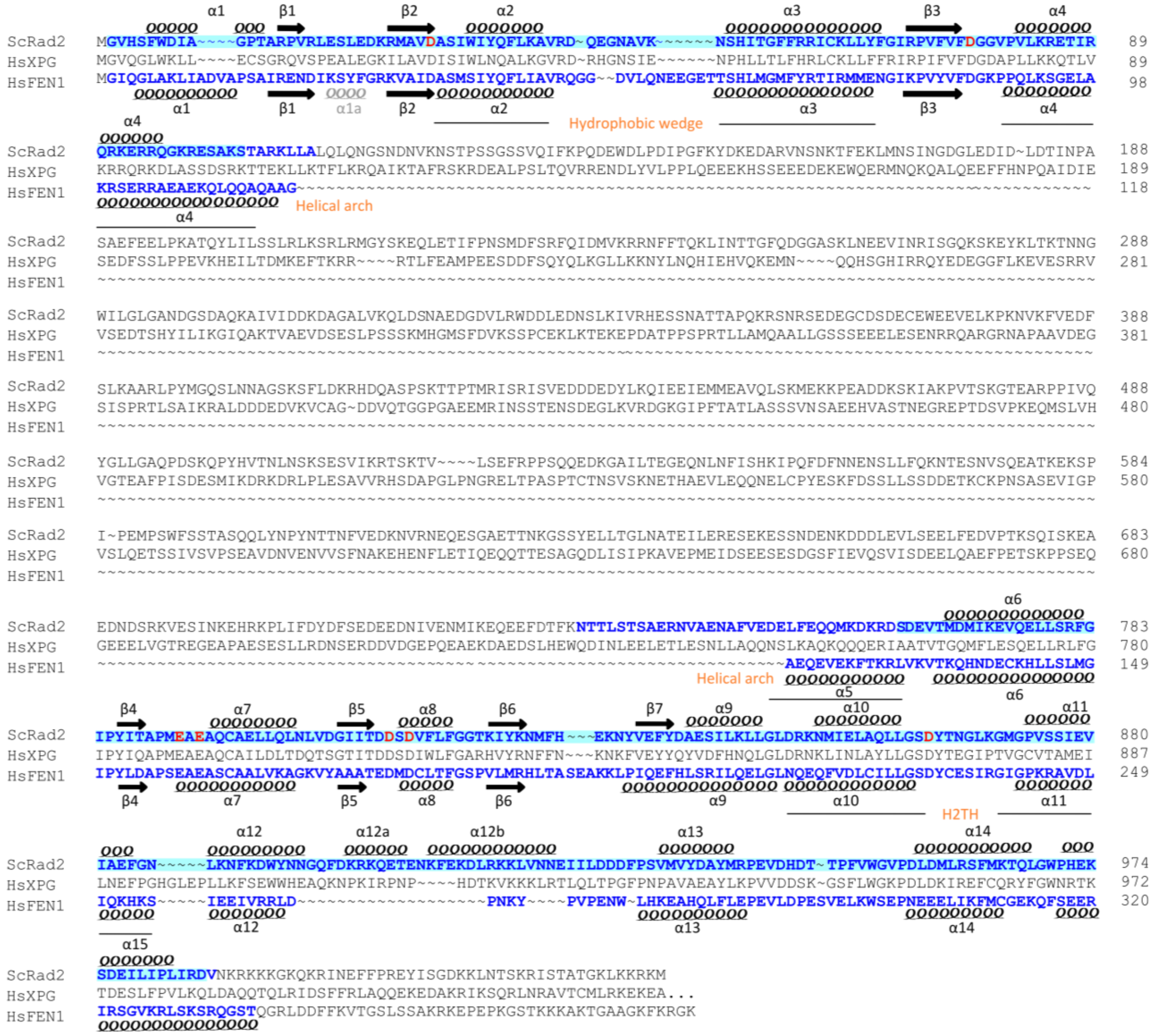

Supplementary Figure 1. Sequence alignment of Sc-Rad2, human XPG and FEN-1. Sc-Rad2 and FEN1 were aligned based on the structures (this work and PDB ID: 3Q8K, respectively). Secondary structure elements for Rad2 and FEN1 are shown and labeled (spirals for helices, arrows for strands). Sequence in blue corresponds to the crystallized constructs. Rad2 residues for which electron density was observed in at least one protein structure are highlighted in cyan. Active site residues are in red. The C-terminal portion of XPG was omitted for clarity.

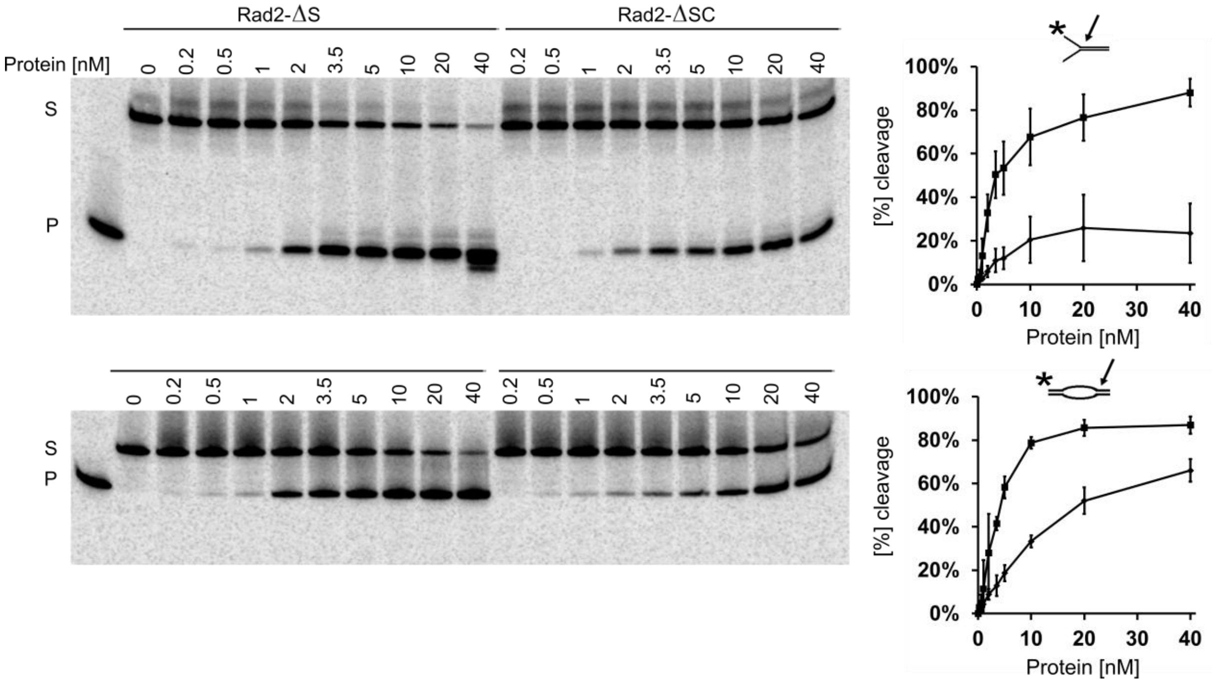

Supplementary Figure 2. Activity of Sc-Rad2 deletion variants on splayed-arm (top panels) and bubble substrates (bottom panels). Protein activity was assayed under the same conditions and at the same protein concentrations as in ref. (10) with substrate at 2.5 nM concentration. The graphs on the right show the results of the densitometry analysis with the plotted percentage of the cleaved substrate for each protein concentration (squares for Rad2-ΔS and diamonds for Rad2-ΔSC). Error bars represent the standard deviation of at least three independent measurements.

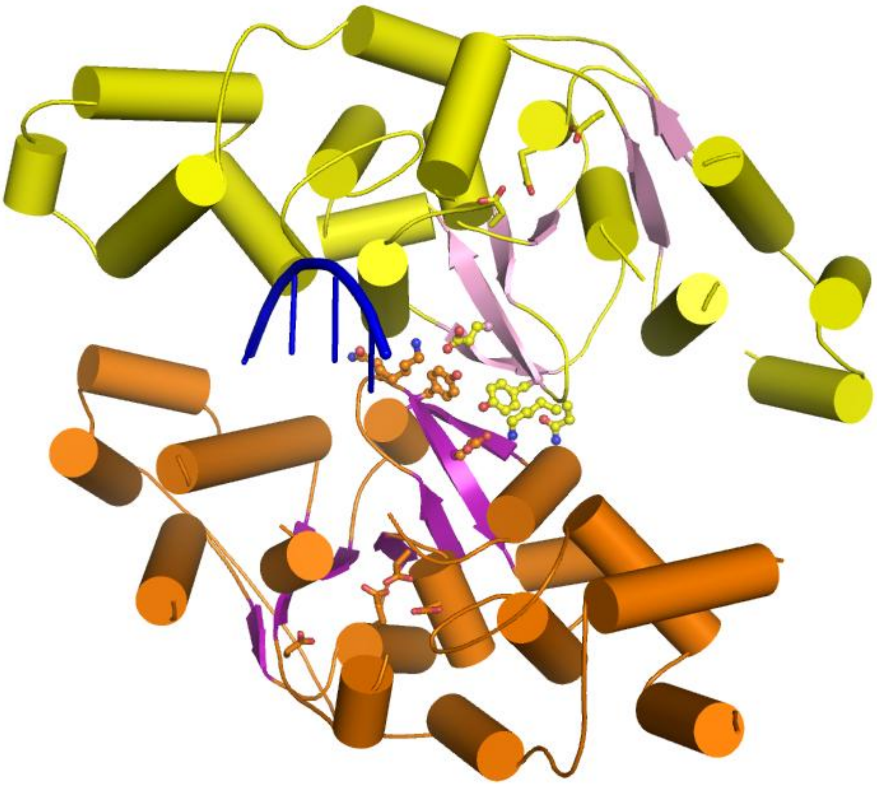

Supplementary Figure 3. Overall structure of Sc-Rad2-ΔSC (complex I). Two dimer subunits are shown in yellow (pink for β-strands) and orange (purple for β-strands). The residues that form the dimerization patch: Lys832, Asn833, Tyr834, and Glu836 are shown with ball-and-sticks, and the active site residues are shown with sticks. A fragment of the DNA observed in the structure is shown in blue.

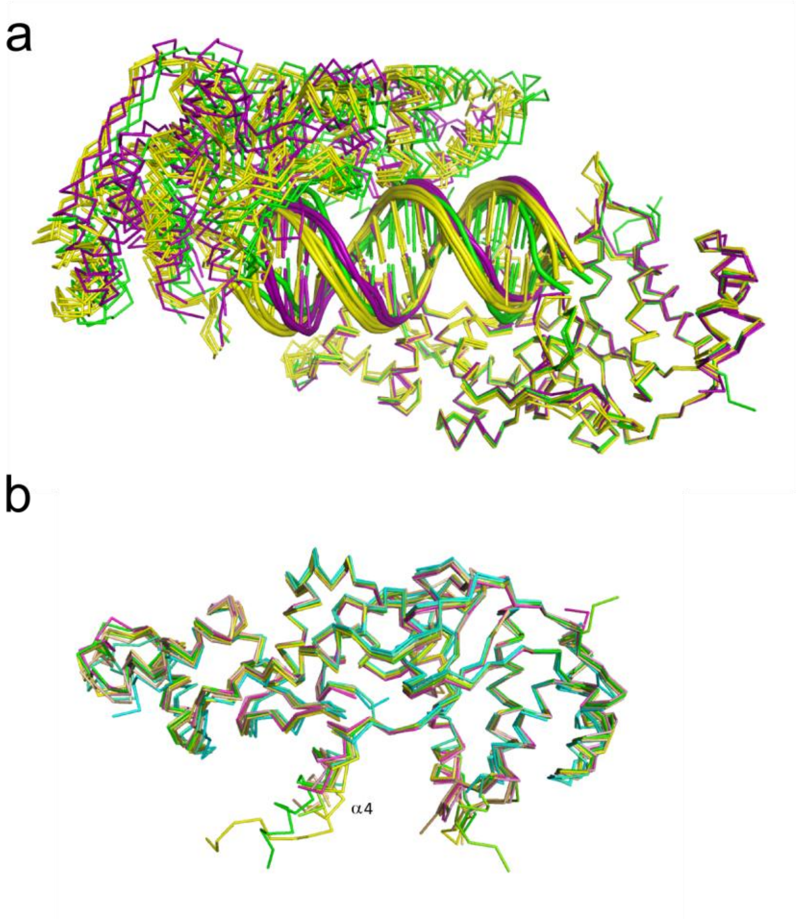

Supplementary Figure 4. Comparison of Sc-Rad2-ΔSC complexes with DNA (a) and Sc-Rad2-ΔSC protein structures (b). Protein is shown in Cα trace (shades of cyan for protein subunits of complex I, green for complex II, yellow for complex III, and magenta for complex IV). Only productive complexes II-IV are shown in (a). Complex structures are superimposed using one protein subunit.

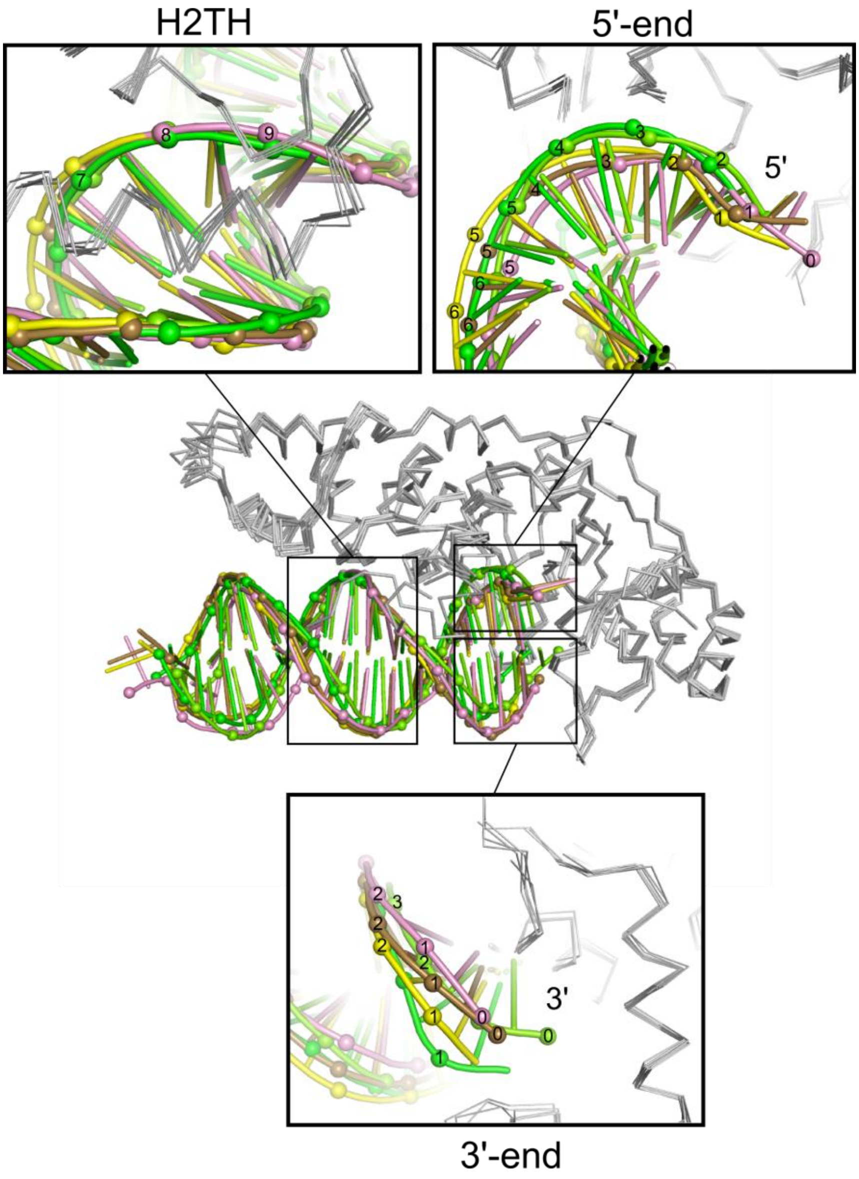

Supplementary Figure 5. Comparison of the conformation of the DNA (complexes II-IV). Protein subunits are shown in gray C-α trace and DNA in cartoon. Different shades of green represent subunits of complex II. Yellow and brown represent two selected subunits of complex III. Pink represents a selected subunit of complex IV. The phosphorus atoms are shown as spheres and numbered. Phosphate 1 belongs to nt +1 (Fig. 2) and is located at the ss/dsDNA junction.

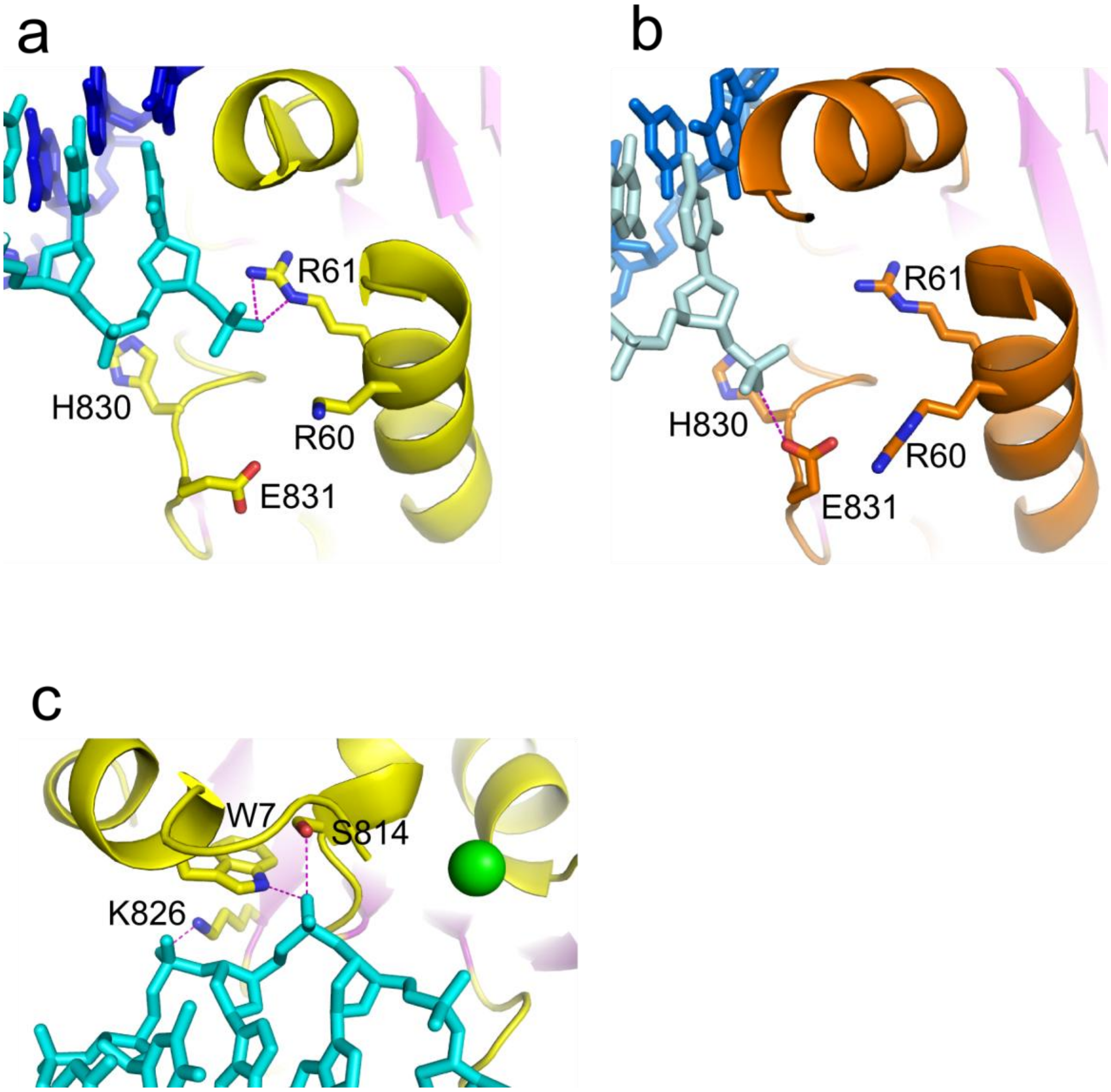

Supplementary Figure 6. Details of the protein-nucleic acid interactions. (a and b) binding of the 3'-phosphate group at the ss/dsDNA junction - complex II (a) and complex III (b). (c) interactions in the vicinity of the active site observed in complex II. The calcium ion at the active site is shown as a green sphere.

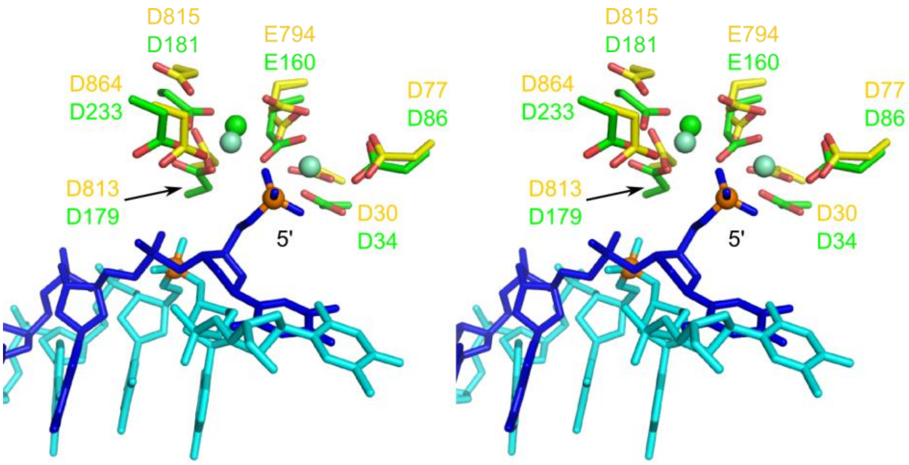

Supplementary Figure 7. Stereoview of a superposition of the active sites of Rad2-ΔSC-DNA complex (yellow for protein and cyan for DNA) and FEN1-product complex (PDB ID: 3Q8K; green for protein and blue for DNA). Metal ions are shown as spheres (green for Ca^{2+} from Rad2 structure and pale green for Sm^{3+} from FEN1 structure). The scissile phosphates are shown as orange spheres.

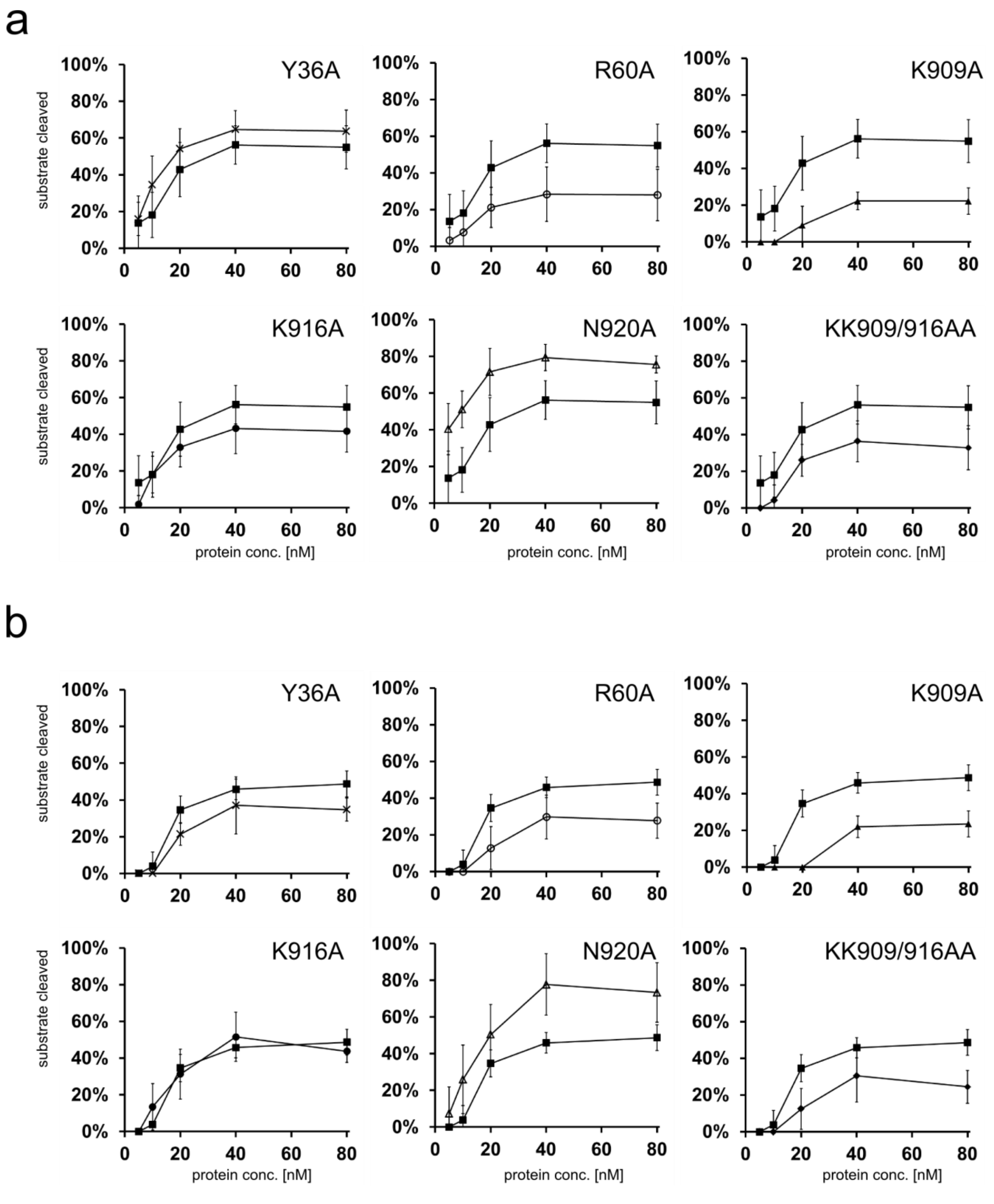

Supplementary Figure 8. Activity of Sc-Rad2-ΔSC variants with point substitutions (densitometry analysis – representative gels are shown in Fig 3). (a) Activity on splayed-arm substrate, (b) activity on DNA bubble substrate. Each data point corresponds to at least three independent measurements. Error bars correspond to standard deviation of each data point. Activity of wildtype protein is plotted in each panel with squares and other symbols are used for variants indicated on top of each panel. Q37A and R61A variant that had virtually no activity are not plotted.

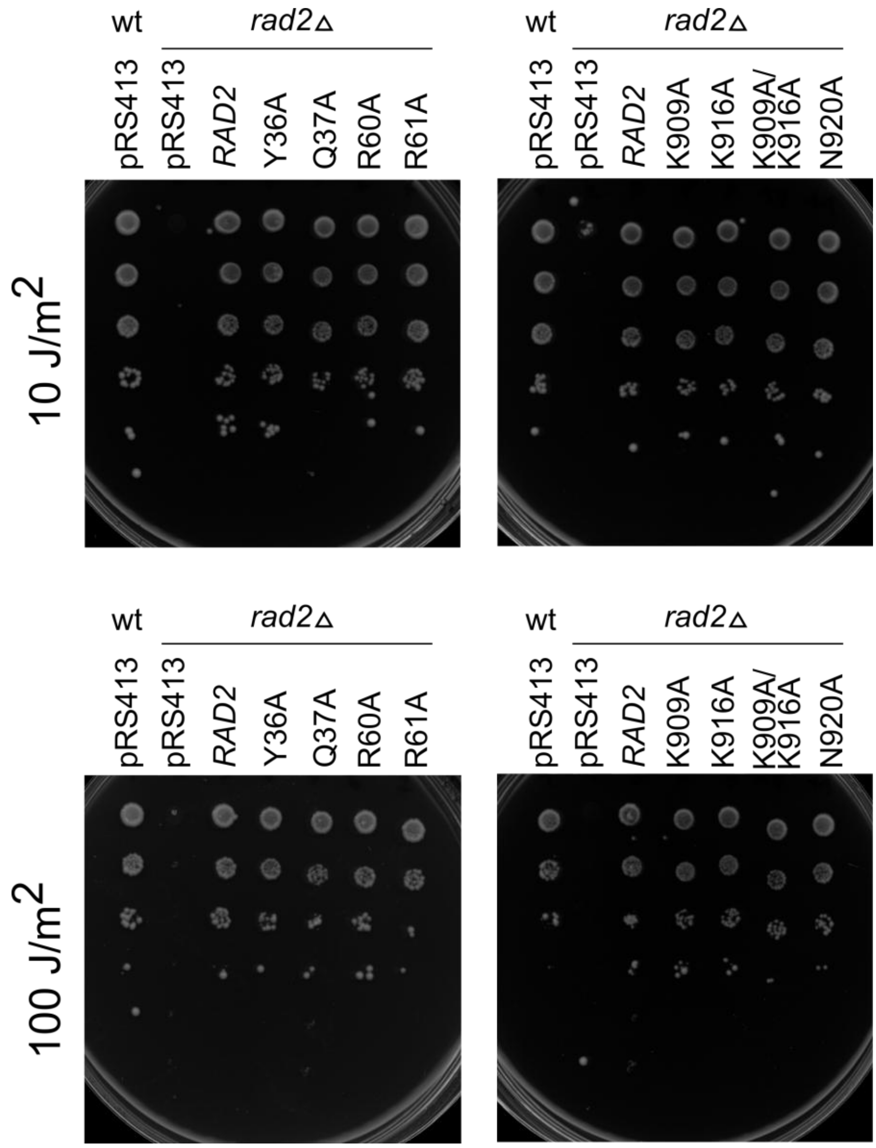

Supplementary Figure 9. Complementation of UV sensitivity. The strains on top of the panels (wild type BY4741 or its counterpart with the deletion of Rad2 gene – Y07289) were transformed with an empty pRS413 vector or pRS413 with the subcloned *RAD2* gene (wildtype or with substitutions indicated on top of each lane). Serial 10-fold dilutions of liquid cultures of the transformants were plated, exposed to the indicated doses of UV light and grown at 28°C.

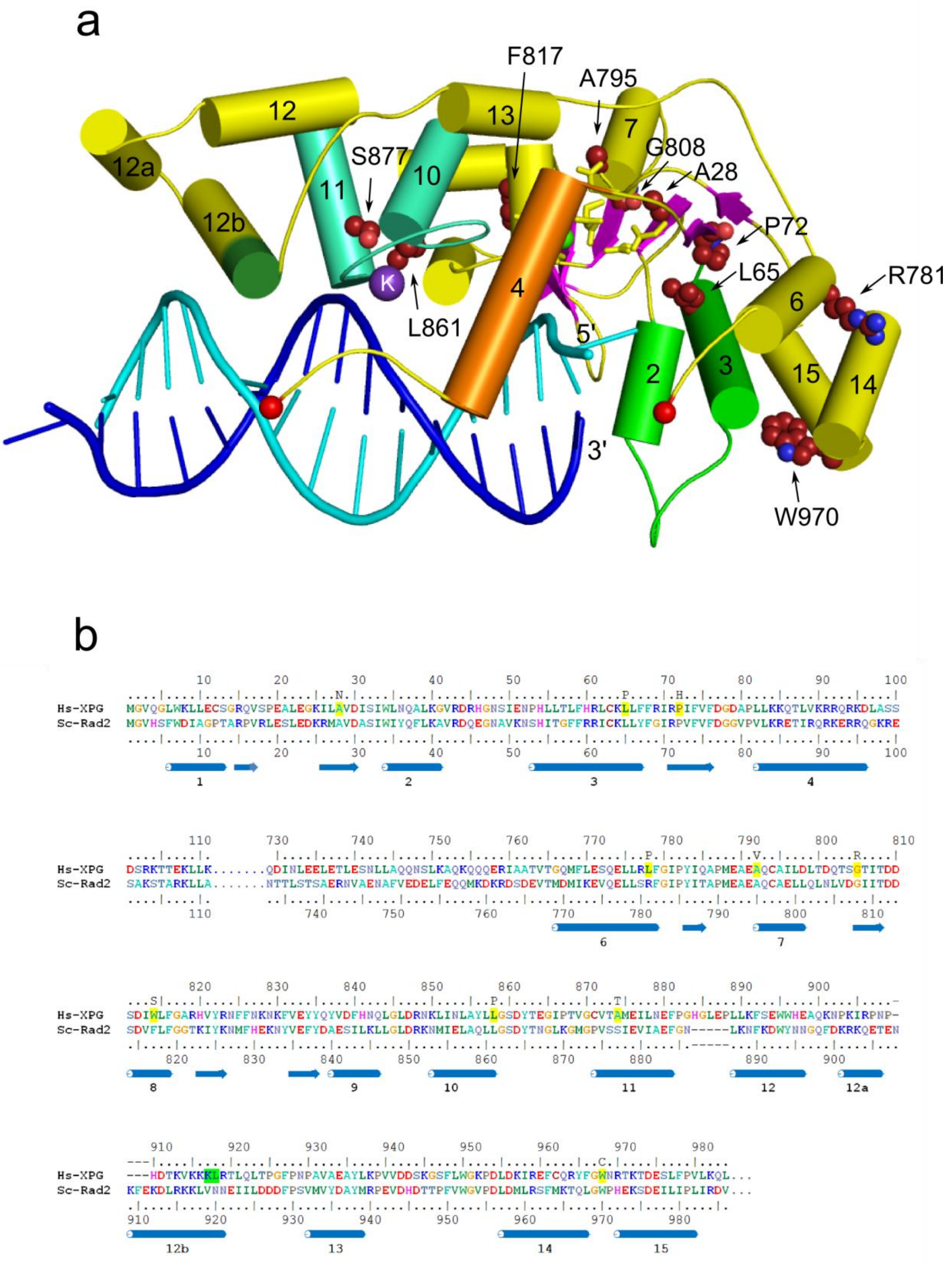
a
F817
A795
12
13
7
G808
A28
12a
S877
10
11
12b
P72
L65
R781
K
L861
4
6
5'
3
2
15
14
3'
W970
b
Hs-XPG
Sc-Rad2

Supplementary Figure 10. Mutations identified in patients (previous page). (a) Structure of Sc-Rad2-ΔSC (only one protein chain is shown for clarity) is colored and labeled as in Figure 1. The equivalents of XPG residues mutated in patients are shown in brown sphere representation and labeled. The site of the 44-residue insertion in helix 12b is marked in dark green. (b) Alignment of N and I segments of human XPG and Sc-Rad2. The regions omitted in alignment are indicated with dots in protein sequence. Sites of point mutations are highlighted in yellow with observed residue substitution on top of the sequence. The site of 44-residue insertion is marked in green. The secondary structures observed in Sc-Rad2-ΔSC are shown as tubes (helices) and arrows (strands). Residues numbers are given.
